# Effects of Isometric Strengthening Exercises on Pain and Disability Among Patients With Knee Osteoarthritis

**DOI:** 10.7759/cureus.18972

**Published:** 2021-10-22

**Authors:** Chinelo N Onwunzo, Sylvester E Igwe, Joseph O Umunnah, Chigozie I Uchenwoke, Uchechukwu A Ezugwu

**Affiliations:** 1 Department of Physiotherapy, Onitsha General Hospital, Onitsha, NGA; 2 Department of Physiotherapy, Coal City University, Enugu, NGA; 3 Physiotherapy, Department of Medical Rehabilitation, Faculty of Health Sciences and Technology, Nnamdi Azikiwe University, Nnewi, NGA; 4 Physiotherapy, Department of Medical Rehabilitation, Faculty of Health Sciences and Technology, University of Nigeria, Enugu, NGA

**Keywords:** knee osteoarthritis, isometric exercise, physiotherapy, pain, disability

## Abstract

Introduction

Osteoarthritis (OA) is the most common form of arthritic disease of the joint worldwide, with the knee joint being the most affected in the body. This study investigated the effects of isometric strengthening exercises on pain and disability among patients with knee osteoarthritis.

Methods

This randomized control trial research design was carried out at the Physiotherapy Departments of Nnamdi Azikiwe University Teaching Hospital, Nnewi, and Chukwuemeka Odumegwu Ojukwu University Teaching Hospital, Amaku, Awka in Anambra State, Nigeria. A total of 40 subjects, nine (22.5%) males and 31 (75.5%) females, were randomly assigned into exercise and control groups. Prior to intervention, the weight and height of each subject were measured. Pain intensity, active range of motion (AROM) and passive range of motion (PROM), and functional ability of both groups were recorded using the Numerical Pain Rating Scale (NPRS), universal goniometer, and Western Ontario and McMaster Universities Osteoarthritis Index (WOMAC), respectively. All participants in the exercise group performed isometric quadriceps and straight leg raise exercises, and the control group received no intervention whatsoever. After six weeks, the pain intensity, AROM, PROM, and functional ability scores were re-measured and documented.

Results

While comparing the pre-test and post-test scores using paired t-test, the exercise group showed a significant difference in each parameter (NPRS, AROM, PROM, and WOMAC = 0.000), while the control group showed no significant difference. Independent sample t-test outcome at six weeks (exercise and control groups) showed significant reduction of pain intensity (NPRS = 0.000), increased range of motion (AROM = 0.000, PROM = 0.003), as well as improvement in function (WOMAC = 0.000) at a significant level of p ˂ 0.05.

Conclusion

At the end of the six weeks, isometric strengthening exercises showed a significant effect on pain intensity, range of motion, and functional ability among subjects with knee osteoarthritis.

## Introduction

Osteoarthritis (OA) is a common disorder characterized by progressive destruction of the articular cartilage in the joint, associated with remodeling of the subchondral bone, synovitis, and formation of bone outgrowths at the joint margins, called the osteophytes. It is also the leading cause of progressive disability [1]. In osteoarthritis, the top layer of cartilage breaks down and wears away, permitting bone under the cartilage to rub together. This rubbing leads to pain, swelling, and loss of motion of the joint. Over time, the joint may lose its normal shape; also bone spurs may grow on the edges of the joint. Bits of the bone or cartilage can break off and float inside of the joint space, which causes more pain and damage [2].

Knee osteoarthritis is a common chronic joint disease and a costly public health problem worldwide [3]. It leads to pain, loss of function, and reduced quality of life [4]. The onset, progression, and severity of knee osteoarthritis have been associated with decreased muscular strength and modifications in joint biomechanics. This leads to fear of movement, which may prevent participation in exercises and social events leading to further physical and social isolation [5]. Osteoarthritis has been ranked one of the most common joint disorders affecting both males and females globally, it affects females more than males [3,6]. Osteoarthritis is one of the most prevalent conditions resulting in disability particularly in the elderly population [7]. The economic impact of knee osteoarthritis is substantial and will further increase as the population ages and obesity rates escalate [8-10]. Globally, as of 2010, approximately 250 million people (3.6% of the population) had osteoarthritis of the knee [11]. A study in a rural community in Nigeria reported that one out of every five Nigerian adults has symptomatic OA of the knee and the majority of them were females [12]. Age, sex, obesity, trauma, joint injuries, gene, bone deformity, and quadriceps (quads) weakness are the risk factors associated with knee osteoarthritis [13-14]. Isometric exercises help to aim at the surrounding muscles of the knee joint.

An isometric exercise is a static form of exercise where muscles contract producing force without any considerable change in the muscle length and without any visible joint movement [15]. Quadriceps strengthening has traditionally been an important component of exercise programs of knee osteoarthritis. This is because quadriceps weakness is the most presented finding among people with knee osteoarthritis [14,16-18], and has been implicated in disease pathogenesis, usually with pain severity, physical dysfunction, and functional decline [19]. The pain and disability caused by osteoarthritis could be worrisome and a thing of great concern to patients and therapists alike.

Though a range of treatment options are available, a good picture efficacy of isometric strengthening exercise on pain and disability in a Nigerian setting is one that has a dearth of available published research resources. The little research that has been carried out possessed a variety in parameters and procedures. As such, therapists rely more on the findings from unrelated geographic locations predominantly Caucasians, which may differ due to environmental factors. There is a need to formulate interventions that are cheap and require little equipment in an environment such as ours in which poverty is rife. Also, there is a need to develop an intervention that can sustain the gains of rehabilitation, prevent the occurrence of pain and disability, and encourage patients to take charge of the treatment. It is in view of the above-mentioned reasons that this work is deemed necessary.

## Materials and methods

To adequately evaluate this research, a randomized control trial research design was used. The study was carried out in tertiary care hospitals, at the outpatient orthopedic unit of the Physiotherapy Departments of the Nnamdi Azikiwe University Teaching Hospital, Nnewi, and Chukwuemeka Odumegwu Ojukwu University Teaching Hospital, Amaku, Awka, all in Anambra State, Nigeria. The research population comprised 40 participants who had symptomatic knee osteoarthritis according to the criteria established by the American College of Rheumatology (ACR) [20], and the participants who met the inclusion criteria gave informed consent for participation. A total of 49 subjects enrolled were screened for inclusion in the study, of those five were deemed ineligible and four did not complete the exercise. The remaining 40 subjects were randomized into two groups (20 in the control group and 20 in the exercise group). A weighing scale was used to measure the weight, measuring tape to measure the height, and active and passive range of motions were measured using a universal goniometer. Data were collected using the Western Ontario and McMaster Universities Osteoarthritis Index (WOMAC), which measured physical function, pain, and stiffness, and the Numerical Pain Rating Scale (NPRS) was used to measure pain intensity. Prior to the study, the age, sex, weight, and height of each subject were measured. Also, pain intensity, active range of motion (AROM) and passive range of motion (PROM), and functional ability of both groups were recorded using NPRS, universal goniometer, and WOMAC, respectively.

All participants in the exercise group performed isometric quadriceps and straight leg raise exercises. The study was performed for six weeks, and each patient was treated for three sessions per week, lasting for about 50 minutes. The first set of exercises was the isometric quadriceps exercise; the second set of exercises was the isometric quadriceps and straight leg raise exercise. The first set of exercises was performed five times a day, with a slow count of one to five in the first three weeks, followed by the two sets of exercises, with initial procedure from the fourth to sixth. The control group received 10 minutes of infrared therapy at the knee joint, with no intervention, and were not visited between assessments and advised to continue with their daily activities.

Active and passive range of motion, Western Ontario and McMaster Universities Osteoarthritis Index (WOMAC), and Numerical Pain Rating Scale (NPRS) were measured at the end of six weeks of exercises for both groups.

Data were analyzed using Statistical Package for Social Sciences (SPSS) version 23 (IBM SPSS Statistics, Armonk, NY), summarized in descriptive statistics of the mean (X) and standard deviation (SD), and were presented in tables. Paired sample t-test was used to compare post-test and pre-test scores for each parameter (AROM/PROM, NPRS, and WOMAC) in each of the two groups. Independent samples t-test was used to compare the mean scores of the baseline and post-test scores of the two groups, at a significant level of p = 0.05.

## Results

A total of 40 subjects comprised of nine (22.5%) males and 31 (75.5%) females (Table 1). The proportion of male and female participants was distributed between exercise and control groups (5:15 and 4:16, respectively). The participants were within the age range of 40-70 years, with a mean age of 58.50 ± 7.75 years. The mean weight, height, and body mass index (BMI) of the participants were 73.80 ± 9.00 kg, 1.62 ± 0.05 m, and 27.95 ± 3.33 kg/m^2^, respectively.

**Table 1:** Comparison of the demographic variables among the two groups. N: number of participants; X ± SD: mean ± standard deviation; kg: kilogram; m: meter; BMI: body mass index.

Variables	Exercise group (N = 20) X ± SD	Control group (N = 20) X ± SD	t-value	p-value
Age (years)	60.30 ± 7.58	56.70 ± 7.66	1.444	0.165
Weight (kg)	71.65 ± 8.33	75.95 ± 9.34	1.600	0.126
Height (m)	1.62 ± 0.50	1.62 ± 0.53	0.487	0.632
BMI (kg/m^2^)	26.96 ± 2.62	28.93 ± 3.71	1.857	0.079
Sex			Frequencies	
Male	5 (25%)	4 (20%)	9 (22.5%)	
Female	15 (75%)	16 (80%)	31 (75.5%)	
Total	20	20		

In Table 2, the paired-samples t-test scores at the baseline and at the end of the study in the experimental group revealed a significant reduction in pain intensity, increased range of motion (ROM), and improved function at p = 0.000, respectively. However, there was no significant change among participants in the control group: WOMAC = 0.062, AROM = 0.083, PROM = 0.069, and NPRS = 0.096.

**Table 2:** Paired-sample t-test assessing for differences in pre-test and post-test among exercise and control groups. X ± SD: mean ± standard deviation; WOMAC: Western Ontario and McMaster Universities Osteoarthritis Index; AROM: active range of motion; PROM: passive range of motion; NPRS: Numerical Pain Rating Scale; *: statistically significant.

Variables	WOMAC	AROM	PROM	NPRS
Exercise group	(X ± SD)			
Week 1	56.75 ± 8.43	92.00 ± 13.80	106.00 ± 12.20	6.05 ± 1.05
Week 6	24.75 ± 5.00	103.75 ± 11.22	117.00 ± 9.65	3.25 ± 0.85
t-value	29.738	8.573	8.904	17.995
p-value	0.000*	0.000*	0.000*	0.000*
Control group				
Week 1	46.85 ± 10.69	91.50 ± 13.09	104.25 ± 12.06	5.35 ± 0.74
Week 6	45.05 ± 9.63	92.25 ± 12.51	107.25 ± 10.45	5.10 ± 0.64
t-value	1.983	1.831	1.928	1.751
p-value	0.062	0.083	0.069	0.096

According to Table 3, the result showed that the means of the two groups (experimental and control groups) of the 40 subjects are statistically significant in all variables (WOMAC = 0.000, AROM = 0.000, PROM = 0.003, and NPRS = 0.000).

**Table 3:** Independent sample t-test comparison of both scores of exercise and control groups after six weeks of intervention. X ± SD: mean ± standard deviation; WOMAC: Western Ontario and McMaster Universities Osteoarthritis Index; AROM: active range of motion; PROM: passive range of motion; NPRS: Numerical Pain Rating Scale; *: statistically significant.

Variables	Exercise group (X ± SD)	Control group (X ± SD)	t-value	p-value
WOMAC	31.95 ± 4.78	9.95 ± 4.29	15.298	0.000*
AROM	11.75 ± 6.12	2.75 ± 2.55	6.062	0.000*
PROM	11.00 ± 5.52	6.00 ± 4.47	3.146	0.003*
NPRS	2.80 ± 0.69	0.45 ± 0.51	12.178	0.000*

## Discussion

In the study, an important observation was recorded that pain intensity reduced in the exercise group but also increased the physical ability in most patients with knee osteoarthritis. Women (75.5%) were almost twice as affected by knee osteoarthritis as the men (22.5%) in the study. The literature has documented that women are more likely to be diagnosed with knee osteoarthritis than men with possible reasons of hormonal changes and extra weight. Studies have shown [3,6] that knee osteoarthritis is one of the most common joint disorders among males and females globally. However, it affects females more than males. Age is one of the leading predisposing factors to osteoarthritis of the knee [21-22]. The age range of 63-70 years formed the bulk in this study, while the rest of the participants were roughly distributed between 49 and 62 years; therefore, as the age increases, the frequency of knee osteoarthritis increases. Also from the study, the prevalence of knee osteoarthritis falls within the weight range 71-80 kg.

The results demonstrated that isometric quadriceps exercise showed a gain in quadriceps muscle strength, reflecting functional performance improvement in the exercise group after the six weeks training program. A study by Shakoor et al. [23] concluded that quadriceps exercise and activities of daily living (ADLs) in patients with knee osteoarthritis beneficially reduced symptoms. Similarly, findings from Anwer and Alghadir [24] show that isometric quadriceps exercises brought significant gains in strength of the quadriceps muscle in the experimental group after the five-week training program.

Our findings for pain perception in this study showed that isometric exercise training of the quadriceps muscles led to pain reduction since individuals tend to report a decrease in physical function when having pain. The studies by Baker et al. [25] and Jebakani et al. [26] corroborate the findings. Though not all the research works cited used the same protocol with this research, yet there was a reduction in pain levels in patients with knee osteoarthritis after the exercise training.

Range of motion (ROM) relates to disability in knee osteoarthritis, which limits movement and external hip rotation [27]. The exercise program increased the ROM in this study both actively and passively at the knee joint of the exercise group participants. This reduction in pain and improvement in function in the exercise group may be attributed to improved quadriceps strength and reduced pain, which therefore lead to an increase in the stability of the knee joint.

Isometric quadriceps exercise has been shown in this study to be effective in increasing the range of motion, leading to good function and a decrease in pain in patients diagnosed with osteoarthritis of the knee. The study by Akodu et al. [28] also recorded an efficacious reduction of pain intensity and functional disability, as well as improvement in knee range of motion in patients with knee osteoarthritis. Therefore, isometric strengthening exercises (static quadriceps and straight leg raise exercises) can be used in the management of patients with knee osteoarthritis. It could also be used as an adjunct while addressing intervention necessary to address related problems to osteoarthritis of the knee.

Limitations

Several limitations exist in this study. Smaller sample size and limited geographic range from which the subjects were recruited may limit the generalized findings. Also, the time interval for the study was limited. However, further studies are needed using a large sample size to determine if similar results can be obtained, especially within and around the geographical region. Further studies are needed to assess the long-term impact of therapeutic exercise in osteoarthritis conditions, as well as a combination of isometric strengthening exercises and other intervention programs or physiotherapeutic modalities for effective relief of these conditions.

## Conclusions

In conclusion, the findings from this study suggest that isomeric strengthening exercises can be an essential management plan for patients presenting with knee osteoarthritis directed toward pain intensity reduction, improving functions of the joints, and facilitating functions relating to activities of daily living. Most essentially, each treatment should be made specifically for each patient.
